# LSD1, a double-edged sword, confers dynamic chromatin regulation but commonly promotes aberrant cell growth

**DOI:** 10.12688/f1000research.12169.1

**Published:** 2017-11-16

**Authors:** Meghan M Kozub, Ryan M Carr, Gwen L Lomberk, Martin E Fernandez-Zapico

**Affiliations:** 1 Genomics Laboratories, Department of Laboratory Medicine and Pathology, Mayo Clinic, Rochester, USA; 2 Schulze Center for Novel Therapeutics, Division of Oncology Research, Department of Oncology, Mayo Clinic, Rochester, USA; 3 Division of Research, Department of Surgery, Medical College of Wisconsin, Milwaukee, USA

**Keywords:** LSD1, KDM1a, histone demethylation, epigenetics, cancer

## Abstract

Histone-modifying enzymes play a critical role in chromatin remodeling and are essential for influencing several genome processes such as gene expression and DNA repair, replication, and recombination. The discovery of lysine-specific demethylase 1 (LSD1), the first identified histone demethylase, dramatically revolutionized research in the field of epigenetics. LSD1 plays a pivotal role in a wide range of biological operations, including development, cellular differentiation, embryonic pluripotency, and disease (for example, cancer). This mini-review focuses on the role of LSD1 in chromatin regulatory complexes, its involvement in epigenetic changes throughout development, and its importance in physiological and pathological processes.

## Introduction

Within the nuclei of all eukaryotic cells, DNA is highly compacted via interactions with histones and numerous other proteins to form chromatin. Histones are involved with supercoiling of DNA and are subjected to post-translation modifications (PTMs) ^1^. Diverse covalent modifications to histones control the structure and dynamics of chromatin and regulate access to DNA, ultimately altering gene expression. Multiple biochemical moieties can be covalently added to specific amino acids on the N-terminus of the histone, or “histone tail” ^1, 2^. The sequence of histone tail modifications, or “histone code”, influences transcription and other processes, including DNA repair, replication, and recombination ^3^.

Histone PTMs were historically thought to be irreversible until the discovery of enzymes catalyzing the addition and removal of methyl groups to lysine and arginine residues on histone tails ^4^. Two evolutionarily conserved families of histone demethylases that recognize H3K4me as a substrate have been identified: lysine-specific demethylases (LSDs) and Jumonji C demethylases (JMJCs) ^5– 7^. LSD enzymes demethylate mono- and di-methyl groups of lysine residues and some non-histone targets. JMJCs belong to the dioxygenase superfamily involved in deoxygenation reactions dependent on ferrous iron and α-ketoglutarate allowing demethylation of trimethylated lysine residues ^6, 7^. Lysine-specific histone demethylase I (LSD1) was first described in 2004, inspiring the hypothesis that histone modification is a highly dynamic process ^8^.

This review focuses on LSD1 (also known as KDM1a) and its role in various physiological and pathological processes. LSD1 acts on histone H3 as a transcription co-repressor through demethylation of lysine 4 (H3K4) or as a transcription co-activator through demethylation of lysine 9 (H3K9) ^7, 9– 11^. The enzyme is essential in the control of wide-ranging biological processes, including cell proliferation ^12^, chromosome segregation ^13^, hematopoiesis ^14^, spermatogenesis ^15^, adipogenesis ^16^, stem cell pluripotency ^17^, and embryonic development ^18^. LSD1 can act as an oncogene, and its overexpression promotes cancer cell proliferation, migration, and invasion ^10, 11^.

## LSD1 complex with regulatory proteins to facilitate histone demethylation

LSD1 consists of three structural domains: N-terminal SWIRM domain, C-terminal flavin adenine dinucleotide (FAD)-binding amine oxidase domain, and the tower domain. The SWIRM domain consists of proteins Swi3, Rsc8, and Moira. Through hydrophobic interactions, the FAD domain closely associated with SWIRM, forming a spherical core. Extending from the core is the tower domain forming an elongated helix-turn-helix motif.

Frequently, LSD1 is found to be associated with a transcriptional co-repressor protein (CoREST) and histone deacetylase (HDAC) 1/2 to form a complex. Interaction with CoREST is necessary for LSD1 H3K4 demethylation ^19– 22^. Association of the FAD domain with CoREST-histone ternary complex results in conformational change, permitting association with the N-terminal H3 tail ^23, 24^. With H3K4 in close propinquity to the FAD domain, oxidative demethylation results in increased affinity for LSD1 ^23, 25– 27^. LSD1 requires the first 20 N-terminal amino acids of the histone tail for substrate recognition and interaction ^28, 29^. The specific amino-acid sequence allows LSD1 to sense the epigenetic messages encoded within histone tail and efficiently carry out demethylation. The presence of other epigenetic marks on H3 has the potential to affect the enzymatic activity of LSD1, suggesting a regulatory role for the H3 tail bereft of all other epigenetic modifications upon LSD1 activity ^29, 30^.

**Figure 1.**
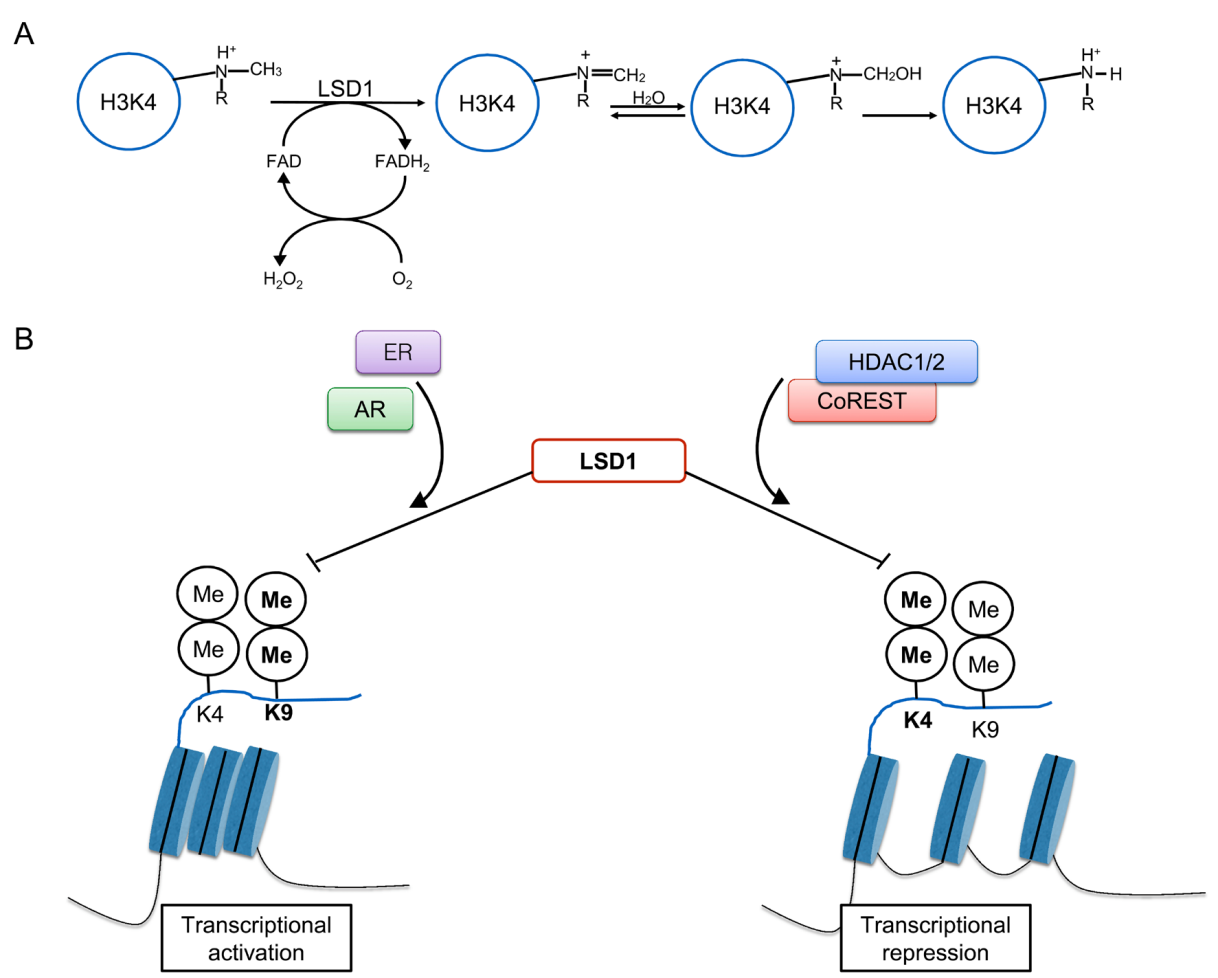
LSD1, through histone 3 demethylation, has dual functions as both a transcriptional activator and repressor. As an illustration of this, LSD1 (lysine-specific demethylase 1) can be associated with activated estrogen receptors (ERs) or androgen receptors (ARs) and promote demethylation at lysine 9 (K9) of the histone tail. This confers opening of heterochromatin, promoting transcriptional activation. Alternatively, LSD1 can complex with CoREST and histone deacetylase 1/2 (HDAC1/2). This association confers more specificity for methylated lysine 4 (K4), resulting in its demethylation. This promotes heterochromatin formation and transcriptional repression.

Within the aforementioned complex, LSD1 demethylates mono- and di-methylated H3K4 ^7, 25, 30, 31^. Earlier studies identified REST as a long-term repressor of neuronal genes in non-neuronal cells ^19, 32– 34^. This was determined to be mediated through the recruitment of the LSD1-CoREST-HDAC complex, thus allowing lysine deacetylation of H3 and H4 in addition to demethylation of H3K4 ^35^. In other investigations, RNA interference (RNAi)-mediated knockdown of LSD1 resulted in H3K4 methylation mark recurrence in proximity to REST target promoters, confirming this regulatory function ^30^. While methylation of H3K4 activates transcription, demethylation by LSD1 confers transcriptional repression ^32^. LSD1 also serves as a transcriptional activator. For example, androgen receptor (AR) activation of its target genes is dependent upon LSD1-mediated H3K9 demethylation ^21^. Following hormone treatment, AR and LSD1 co-localize to promoters, resulting in H3K9 demethylation without changing H3K4 methylation status ^9^. As expected, LSD1 knockdown resulted in reduced activation of AR-responsive promoters ^7, 35^. Taken together, the above findings showed the potential of LSD1 as a transcriptional repressor or activator through dynamic and selective H3K4 demethylation and H3K9 demethylation, respectively ( Figure 1).

## LSD1 contributes to broad, dynamic gene regulation through histone demethylation

Under physiological conditions, LSD1 has been found to have several functions ranging from regulation of hormone receptor–mediated transcription, appropriate hematopoietic stem cell differentiation, and cell cycle control. Estrogen receptor (ER)-mediated transcription is driven by LSD1-mediated H3K9 demethylation at both the gene promoter and an upstream enhancer site. ER then takes part in bending chromatin to interact with RNA polymerase to promote transcription. LSD1 co-localizes to this DNA loop, and the process of demethylation results in formation of hydrogen peroxide, inducing local oxidative DNA damage, recruiting 8-oxoguanine-DNA glycosylase 1 and topoisomerase IIβ. These enzymes induce DNA conformational changes necessary for efficient promotion of gene expression ^36, 37^. Thus, LSD1 plays an important role in hormone receptor–mediated gene expression via histone demethylation and through DNA damage–induced chromatin remodeling.

In addition to driving hormone receptor–mediated gene expression, LSD1 plays a critical role in hematopoiesis. LSD1 is dynamically involved in hematopoietic differentiation through cooperation with growth factor–independent (GFI) proteins. GFI proteins promote expression of lineage-specific genes, and LSD1 specifically interacts with GFI1B binding sites. In mouse models, RNAi depletion of LSD1 impairs both erythrocyte and megakaryocyte differentiation but activated spontaneous granulocyte differentiation. Therefore, actions of LSD1 may be lineage-specific ^38^. However, alternative models have revealed competing findings that LSD1 is necessary for terminal differentiation of erythroid, megakaryocytic, and granulocytic lineages ^38– 40^. Although its specific role is not fully characterized, LSD1 was found to regulate promoters and enhancers of genes associated with hematopoietic stem cells and was critical for terminal differentiation of mature hematopoietic cells as LSD1 knockout resulted in severe pancytopenia ^41^.

Finally, LSD1 biological functions are associated with the regulation of the methylation status of non-histone proteins. Studies demonstrate the relationship between retinoblastoma gene ( *RB1*) and LSD1 in cell cycle control. Overexpression of RB1, the first identified tumor suppressor, ** causes cells to undergo arrest in the G _1_ phase of the cell cycle, and, as expected, abrogation of RB1 ** accelerates G _1_ transition ^42, 43^. Phosphorylation is a key mechanism by which RB1 is regulated. Dephosphorylation is mediated by myosin phosphatase, which promotes cell cycle arrest ^44^. Interestingly, myosin phosphatase target subunit 1 (MYPT1) was identified as a novel substrate of LSD1. Methylation of MYPT1 is mediated by LSD1, preventing dephosphorylation of RB1 and ultimately promoting cell cycle progression ^12^. Given these diverse functions, one can see how altered LSD1 activity could contribute significantly to normal homeostasis and pathology such as malignancy.

## Aberrant LSD1 activity suppresses cell cycle regulators and promotes tumor growth

Given the aforementioned role of LSD1 in cell cycle regulation, one may hypothesize that it could serve as an oncogene in the context of malignant transformation. LSD1 was first found to be overexpressed in neuroblastoma, correlating with poor differentiation ^45^. Overexpression of LSD1 has been documented in many solid tumors and is correlated with aggressive clinicopathological features and poor patient outcomes ^11, 46– 49^. Both *in vitro* and *in vivo* models have demonstrated overexpression of LSD1 correlating with significant chromatin modifications and malignant transformation ^50, 51^. Both pharmacological inhibition and genetic depletion of LSD1 have been shown to inhibit cancer cell proliferation, differentiation, invasion, and metastasis in animal models ^7, 40, 52^. Thus, LSD1 has been confirmed to be an important oncogenic driver, a potential biomarker indicative of poor prognosis, and a potential therapeutic target. There are several forms of malignancy that have been shown to have aberrant LSD1 activity.

First, LSD1 is critical in the process of terminal differentiation in hematopoiesis, and abnormal LSD1 activity is correlated with a variety of myeloproliferative disorders ^11, 50, 51, 53^. Many studies propose LSD1 as a prospective treatment target for acute myeloid leukemia (AML). AML is a heterogenous hematopoietic malignancy characterized by the accumulation of incompletely differentiated progenitor cells (blasts) in the bone marrow, causing suppression of normal hematopoiesis ^41^. LSD1 is a required constituent of a mixed-lineage leukemia (MLL) super complex associated with active transcription sites ^54^. Abrogation of LSD1 results in heightened rates of apoptosis and impaired leukemogenicity in an MLL-AF9 mouse model ^55^. In acute promyelocytic leukemia, all- *trans* retinoic acid can induce differentiation of leukemic cells whereas AML is not responsive. However, inhibition of LSD1 activity in AML models results in increased H3K4me2 at myeloid differentiation–associated genes, resulting in increased responsiveness to all- *trans* retinoic acid ^40^. These data support the importance of LSD1-mediated alteration of the leukemic epigenome in pathogenesis and demonstrate how the enzyme could serve as a therapeutic target in AML.

LSD1 is also dysregulated in solid tumors, including colorectal carcinoma. Increased activity in colon cancer is associated with increased metastatic potential ^56^. Higher expression of LSD1 and low expression of CDH-1 (E-cadherin) in colorectal cancer were associated with higher tumor-node-metastasis staging and thus poorer prognosis ^57^. Knockdown of LSD1 results in CDH-1 upregulation and confers reduced invasiveness. LSD1 regulates the *CDH-1* promoter and demethylation of H3K4me2 causes downregulation of CDH-1 expression ^58^.

Squamous cell carcinoma (SCC) is also associated with elevated LSD1 activity. The most common genetic aberration in SCC of the lung, esophagus, and oral cavity is amplification of *Sox2* ^59– 61^. *Sox2* encodes a transcription factor important in embryonic stem cells with the ability to reprogram somatic cells into induced pluripotent stem cells. Elevated LSD1 levels are associated with amplified *Sox2* expression in lung cancer. Cells from these patients are particularly sensitive to the LSD1 inhibitor, CBB1007, whereas Sox2-negative cells are not ^62^. Subsequent chromatin immunoprecipitation (ChIP) sequencing revealed that LSD1 binds to the *Sox2* gene and is enriched in the transcriptional regulator region, a known distant enhancer for Sox2 expression in breast cancer. LSD1 inhibition revealed that its activity is required for Sox2 expression. Inactivation results in increased global H3K9me1/me2 and H3K27me3 with formation of bivalent chromatin domains within the regulatory regions of *Sox2* and cell cycle regulatory genes, leading to suppression of gene expression ^63^.

ER-negative breast cancer is a subtype of the common malignancy with relatively more rapid growth, loss of differentiation, and increased propensity for metastasis. Interestingly, LSD1 tends to be highly expressed in this form of breast cancer ^64^. LSD1 and HDAC closely interact and control the growth of breast cancer through aberrant gene silencing ^65^.

## Upregulation of LSD1 promotes epithelial-to-mesenchymal transition

Aberrant LSD1 activity is extensively characterized in multiple cancers, but the mechanism by which it promotes cancer progression extends beyond suppression of cell cycle regulators. For example, through PTM of a notable non-histone protein, p53, LSD1 represses apoptosis. This is achieved through demethylation of K370me2. Whereas methylation at this site promotes association of p53 with co-activator 53BP1, LSD1 inhibits this interaction ^66^. A more thoroughly studied role of LSD1 is in the epithelial-to-mesenchymal transition, a critical process in cancer progression. Snail and Slug are key molecular mediators of epithelial-to-mesenchymal transition through direct repression of epithelial markers such as CDH-1. This is achieved through the SNAG domain of Snail, structurally resembling the histone H3 tail, recruiting LSD1 to epithelial gene promoters with formation of the Snail-LSD1-CoREST complex with subsequent demethylation of H3K4me2 ^67, 68^. In the specific case of neuroblastoma, MYCN has been correlated with poor prognosis. This is related to the co-localization of LSD1 and MYCN at the promoter of a key suppressor of metastasis, N-Myc downstream-regulated gene 1 (NDRG1), inhibiting its expression. Thus, elevated LSD1 is associated with lower NDRG1 expression and poor prognosis ^69^. Inhibition of enzymatic activity or abrogation of the SNAG-LSD1 interaction suppresses mesenchymal markers, decreasing cancer invasiveness ^70, 71^. Promoting transition to a mesenchymal phenotype is opposed by acetylation of LSD1 by males absent on the first (MOF). In fact, MOF expression correlates with favorable prognosis in cancer ^72^.

In summary, there is an abundance of evidence for a role of LSD1 in the pathogenicity of a wide array of malignancies. Cancer is the consequence of complex and heterogeneous genetic alterations, and aberrant LSD1 activity can contribute to a malignant phenotype through extensive modifications of the epigenome. Therefore, therapeutic targeting of the demethylase may prove an effective strategy in reversing or attenuating more aggressive malignant phenotypes in many cancers.

## Conclusions

This review has briefly summarized the current knowledge and research of LSD1, its expression patterns, recruitment mechanisms, chromatin remodeling, biochemical functions, molecular structure, and role in cancer. In the past several years, studies have elucidated the role that histone lysine demethylases play in epigenetic regulation. LSD1 was the first histone demethylase identified and catalyzes the oxidation of methylated H3K4 through an amine oxidation reaction. Furthermore, LSD1 can act on non-histone proteins. While LSD1 is critical in conferring the dynamic nature of epigenetic regulation through histone modification, imbalance in histone modification with excessive LSD1 activity is significantly associated with increased cellular growth and suppression of cell cycle regulatory proteins in a broad array of tissues. Thus, LSD1 represents a critical oncogene and potential therapeutic target. More is to be elucidated regarding the function of LSD1. Interestingly, Wang *et al*. reported that LSD1 inhibits the invasion of breast cancer cells *in vitro* but conversely suppresses breast cancer metastatic potential *in vivo* ^73^. These data suggest not only that LSD1 is multifunctional but also that its functions may be highly context-dependent. More extensive studies into the effects of these contexts on LSD1 function will be important to fully understand its role in cancer. A significant amount of research has been devoted to the development of a wide range of epigenetic therapies. Numerous questions still must be answered regarding LSD1 if effective therapeutic strategies are to be developed. For example, what are the functions and interactions of other LSD1 domains, or does LSD interact with methyltransferases, and how are these relationships regulated? In regard to the development of further therapies, emphasis should be placed on the development of highly specific drugs for demethylase subtypes to better direct the desired epigenetic effect of the drug. This would help specifically discern enzyme subtype mechanism. Despite these challenges, LSD1 is clearly important in normal cellular functions and malignancy.
